# Correction to “Prognostic Significance of the Holter‐Derived T‐Wave Variability in Patients With Ventricular Tachyarrhythmias Complicating Acute Coronary Syndrome—TWIST Study”

**DOI:** 10.1111/anec.70046

**Published:** 2024-12-20

Makino, T., Ichikawa, T., Amino, M., Nakamura, M., Koshikawa, M., Motoike, Y., Nomura, Y., Harada, M., Sobue, Y., Watanabe, E., Kiyono, K., Yoshioka, K., Ikari, Y., Ozaki, Y., & Izawa, H. (2023). Prognostic significance of the Holter‐derived T‐wave variability in patients withventricular tachyarrhythmias complicating acute coronary syndrome—TWIST study. Annals of Noninvasive Electrocardiology, 28, e13069. https://doi.org/10.1111/anec.13069

The new affiliation has been added to the first coauthor Taro Makino and the affiliation detail is shown below.

Taro Makino MD^1,4^

^4^Department of Cardiology, Fujita Health University School of Medicine, Toyoake, Japan

We apologize for this error.

